# Head movements and postures as pain behavior

**DOI:** 10.1371/journal.pone.0192767

**Published:** 2018-02-14

**Authors:** Philipp Werner, Ayoub Al-Hamadi, Kerstin Limbrecht-Ecklundt, Steffen Walter, Harald C. Traue

**Affiliations:** 1 Neuro-Information Technology group, Institute for Information Technology and Communications, Otto-von-Guericke University Magdeburg, Magdeburg, Germany; 2 Department of Anesthesiology, University Medical Center Hamburg-Eppendorf, Hamburg, Germany; 3 Medical Psychology, University Clinic for Psychosomatic Medicine and Psychotherapy, Ulm, Germany; Taipei Veterans General Hospital, TAIWAN

## Abstract

Pain assessment can benefit from observation of pain behaviors, such as guarding or facial expression, and observational pain scales are widely used in clinical practice with nonverbal patients. However, little is known about head movements and postures in the context of pain. In this regard, we analyze videos of three publically available datasets. The BioVid dataset was recorded with healthy participants subjected to painful heat stimuli. In the BP4D dataset, healthy participants performed a cold-pressor test and several other tasks (meant to elicit emotion). The UNBC dataset videos show shoulder pain patients during range-of-motion tests to their affected and unaffected limbs. In all videos, participants were sitting in an upright position. We studied head movements and postures that occurred during the painful and control trials by measuring head orientation from video over time, followed by analyzing posture and movement summary statistics and occurrence frequencies of typical postures and movements. We found significant differences between pain and control trials with analyses of variance and binomial tests. In BioVid and BP4D, pain was accompanied by head movements and postures that tend to be oriented downwards or towards the pain site. We also found differences in movement range and speed in all three datasets. The results suggest that head movements and postures should be considered for pain assessment and research. As additional pain indicators, they possibly might improve pain management whenever behavior is assessed, especially in nonverbal individuals such as infants or patients with dementia. However, in advance more research is needed to identify specific head movements and postures in pain patients.

## Introduction

Pain is a personal experience with behavioral response like verbal report, display of nonverbal behavior such as crying and moaning, facial expression, or body language. Objective assessment of such pain behaviors can complement pain diagnosis based on self-report and can replace spoken reports for individuals who cannot communicate their distress verbally, e.g. infants or adults with severe cognitive deficits [1, 2, 3, 4]. Next to classical observation, efforts were made in the attempt to create automated diagnostic tools [5, 6, 7, 8, 9, 10].

Several observational scales have been developed to assess pain, e.g. the COMFORT scale [11], FLACC [12], CNPI [13], BPS [14], CPOT [15], PAINAD [16], and PACSLAC [17]. All considered scales assess facial expression, body movements, and acoustic indicators. Typical body language clues that are associated with pain include guarding, touching or rubbing the affected area, restlessness, and muscle tension. Body movements serve purposes of escape or avoidance of threat and are capable of eliminating or ameliorating painful experience. Observers can use this information for diagnostic purposes; observational scales attempt to systematize that. For details and discussion on pain assessment tools the reader is referred to review papers [3, 18, 19, 20, 21].

This article focuses on head movements and postures. Outside the context of pain, they are known to play a considerable role in social interaction and nonverbal communication. We turn our head towards a conversational partner, nod to indicate understanding and agreement, and use additional gestures to indicate dissent, confusion, or consideration [22]. The head can direct another person’s visual attention. If the gaze of a person is shifting, it is often towards an object of interest. Even 6-month-old infants exploit this property by following the gaze of their caregiver [23, 22]. Further, attention can be guided intentionally by exaggerated head movements like pointing with a finger [22]. Head movements are involved in behavioral mirroring and mimicry [24, 25]. Head orientation also communicates emotion. The lateralization hypothesis of the brain hemispheres predicts more right oriented head movement, since processing of emotions generally—and especially of negative emotions—is located in right cortical hemisphere [26]. According to Mignault and Chaudhuri, a bowed head connotes submission, inferiority emotions (as shame, shyness, regret, guilt, and embarrassment), and sadness; a raised head connotes dominance, superiority emotions (as pride, self-assurance), joy, and contentment [27]. Wallbott found disgust and shame to be associated with a downward head, and joy, pride, and boredom to be associated with a raised head [28]. Other studies confirm that a bowed head is part of the typical display of embarrassment [29, 30].

The clinical population of depression patients shows altered head movement behavior. Several studies found less and slower head movement [31, 32] and more downward head postures [33, 31] compared to healthy controls or to the time after successful treatment.

In the context of pain assessment, only few works considered head movements and postures so far, therefore there is a research gap in this area. Only one of the reviewed behavioral pain scales mentions the head explicitly: The COMFORT scale describes the highest rating of the “Physical Movement” item as “vigorous movements including torso and head” [11]. Further, resting head posture has been studied for some clinical populations, showing that people with neck pain [34] and cervical headache [35] show forward bended head postures possibly in order to relax neck muscle tension or to relief cervical joint pressure. Testing the hypothesis of emotional inhibition as an etiological factor for muscle tension, Traue et al. found that tension headache and back pain are associated with reduced head motion [36, 37].

Our study is motivated by results of Werner et al., who analyzed the facial response to heat pain through computer vision and statistical learning techniques [5, 38, 39]. Based on observed head movements only, the statistical model could predict whether an unseen video was recorded during a painful stimulus or during rest in more than 65% of the cases, which is highly significantly above chance. These results suggest that specific head movements might be valuable additional indicators in pain assessment. The works of Werner et al. [5, 38, 39] have several limitations, which we address here. First, they apply black-box machine learning and do not identify the specific head movements that the learned model uses to distinguish pain and control trials. Second, their work does not include appropriate background information, statistical analysis, and discussion that are expected by the medical and psychological pain research community. Third, they only analyze one dataset, whereas evidence from multiple datasets would strengthen the results and the conclusions. Based on the hypothesis that specific head postures and movements and pain are related, we analyze three pain datasets aiming at unveiling the potential role of head postures and movements in pain assessment in general. In this initial research we do not focus on specific patient groups, but analyze the available datasets, which comprise behavioral reactions to active pain stimuli of healthy adults and shoulder pain patients. The results of all three datasets support the hypothesis that pain and head movements are related.

## Methods

### Datasets

To validate head movements and postures that differentiate between painful and other situations, we analyze three publically available pain research datasets: the BioVid Heat Pain Database (BioVid) [40, 39], the UNBC-McMaster Shoulder Pain Expression Archive Database (UNBC) [41, 42], and the BP4D-Spontaneous Database (BP4D) [43]. An overview on the datasets is given in Table 1. To the best of our knowledge, these are the only datasets that are available and suitable for analyzing head movements in the context of pain.

**Table 1:** Overview on analyzed datasets. In each dataset, the same subjects underwent painful trials and control trials. For the BioVid dataset, several videos were excluded from analyses, because participants left the camera’s field of view or visual review revealed obvious pose measurement errors. Abbreviations: M = mean, SD = standard deviation.

	BioVid (Part A)	UNBC (available part)	BP4D
**subjects**	87	25	41
- female/male	43/44	13/12	23/18
- age (years)	range: 20–65; M (SD): 41.2 (14.6)		range: 18–29
- population	healthy	shoulder pain patients	healthy
**painful trials**	heat (temperature at pain tolerance) at right forearm	range-of-motion tests with affected limb	cold pressor test with left arm
- videos/trials (count)	1708 of 1740	109	41
- video duration (seconds)	5.5	range: 4.1–27.3; M (SD): 11.8 (4.3)	range: 8.5–65.6; M (SD): 43.6 (18.1)
**control trials**	no heat, rest in between pain stimuli (not painful)	range-of-motion tests with unaffected limb (less painful)	7 emotion elicitation tasks (not painful)
- videos/trials (count)	1723 of 1740	91	7*41 = 287
- video duration (seconds)	5.5	range: 1.9–16.2; M (SD): 7.2 (2.5)	range: 1.6–132.8; M (SD): 44.8 (24.5)

#### BioVid Heat Pain Database (BioVid)

The BioVid Heat Pain Database [40, 39] was collected in a study with 90 participants aged 20 to 65 years. Pain was induced experimentally by a Medoc PATHWAY Advanced Thermal Stimulator (Medoc Ltd., Ramat Yishai, Israel) at the right arm (posterior forearm near the wrist). The participants were sitting on a chair with the arms resting on a desk in front of them. The experiments were recorded with video cameras and physiological sensors (ECG, EMG, and SCL). The participants were explicitly allowed to move their head freely, i.e. there was no instruction to look towards the camera. To reduce the influence of social factors on pain behavior, the experimenter left the room during the main pain stimulation parts (still being contactable for the participant). For our analysis we use Part A of the dataset. It comprises videos of 87 participants; for each of them there are 20 non-painful trial videos (control trials), which were recorded in between the pain stimuli, and 20 trial videos of pain behavior during heat stimulation at the person-specific pain tolerance level. The individual tolerance of each participant had been determined in advance. For more details, the reader is referred to [40, 39] and the BioVid website (http://www.iikt.ovgu.de/BioVid.html).

#### UNBC-McMaster Shoulder Pain Expression Archive Database (UNBC)

Prkachin and Solomon conducted a study with 129 patients who suffered from shoulder pain [42]. The participants underwent several active and passive range-of-motion tests to their affected and unaffected limbs. Tests were performed on both the affected and the unaffected limb to provide a within-subject control. For each test, the subject rated the maximum experienced pain on a visual analog scale (VAS). Facial reactions were videotaped and analyzed [42].

Later, Lucey et al. selected a part of the original dataset (200 trials of 25 participants), named it “UNBC-McMaster Shoulder Pain Expression Archive Database”, and made it available for researchers who work on perception of pain expression or on improved clinical assessment methods [41]. We use this dataset to study the head movement during pain and split it based on the test side. As to be expected, the range-of-motion tests with the affected limb were more painful (VAS mean M = 4.8, standard error SE = 0.3) than with the unaffected limb (control trials), which however were still painful in several cases (M = 1.4, SE = 0.3). More details can be found in [42, 41].

#### BP4D-Spontaneous Database (BP4D)

To collect the BP4D-Spontaneous Database (BP4D) [43], each of 41 subjects was videotaped while performing eight tasks that were meant to elicit spontaneous (not posed) facial expressions. One of these tasks (T6) was a cold pressor test [44] to induce pain, i.e. the subject submerged his left arm into ice water. We use the other tasks, which were meant to elicit emotion, as control trials: (T1) the participant talked to the experimenter and listened to a joke to elicit happiness or amusement, (T2) the participant watched a documentary about a real emergency involving a child and discussed it with the experimenter to elicit sadness, (T3) the participant heard a sudden, unexpected burst of sound to elicit surprise or startle, (T4) he had to improvise a silly song to elicit embarrassment, (T5) the participant played a game that occasioned physical threat to elicit fear or make him nervous, (T7) following the cold pressor test, the subject experienced harsh insults from the experimenter to elicit anger, and (T8) he experienced an unpleasant smell to elicit disgust. The experimenter was a professional actor and director of performing arts. Self-report of the participants suggested that the emotion elicitation was successful, whereas (T7), next to anger, also elicited embarrassment very often. Although the database was primarily designed to elicit facial expression, we only use it to analyze head postures and movements occurring during the tasks.

### Measurement of head movements and postures

**Fig 1:**
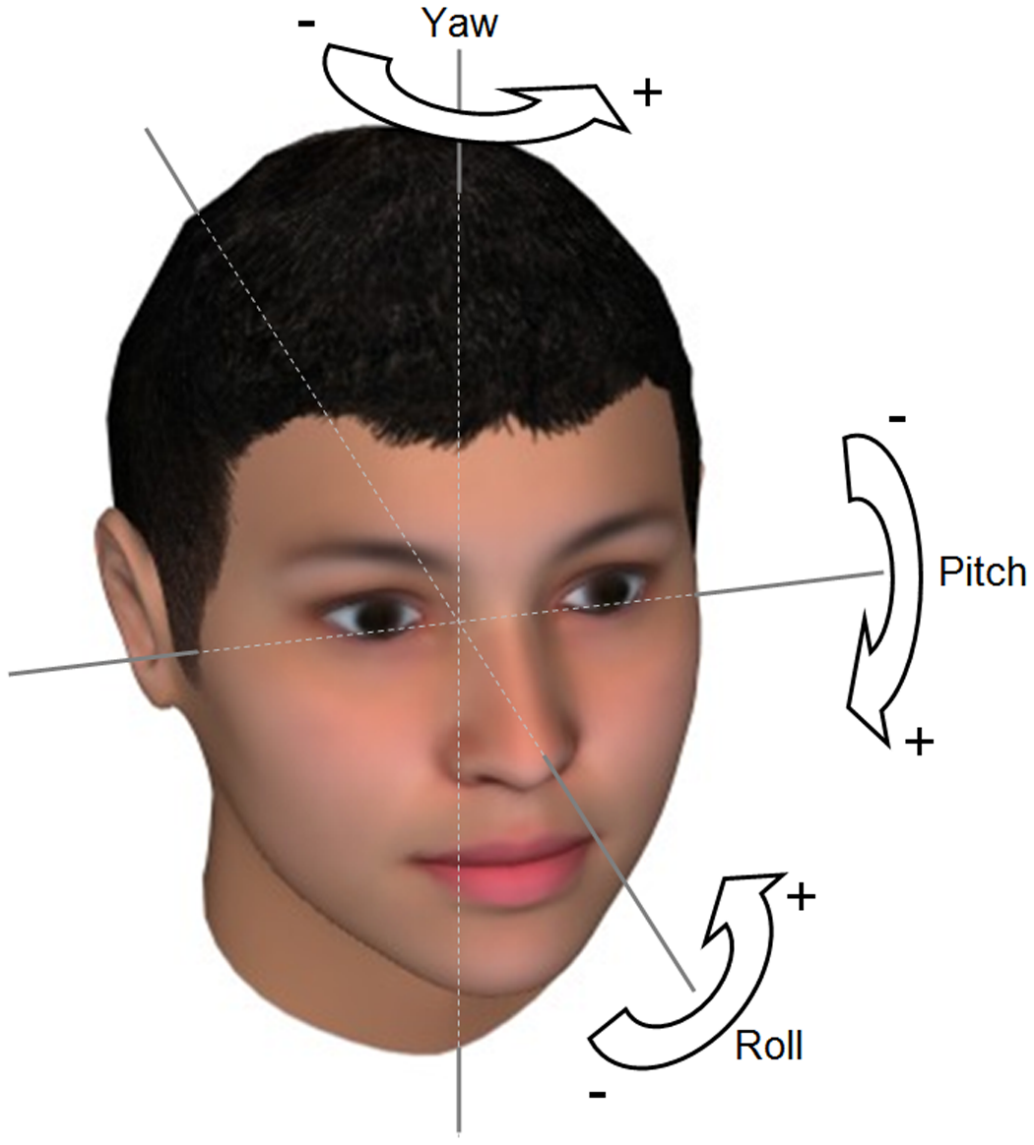
Egocentric rotation angles describing orientation of the head in degrees (DEG). Pitch quantifies down- or upward head orientation, yaw quantifies left or right head turn, and roll quantifies right or left head tilt.

A head movement is a sequence of head postures (which are also called head poses). A head pose can be described by the head’s position and orientation in the 3-dimensional space. Many of the works on head pose (including this work) focus on the orientation [22]. It can be characterized by three egocentric rotation angles named pitch, yaw, and roll (see Fig 1). The pitch angle quantifies the up- or downward head orientation (downward with positive values), the yaw angle quantifies the left or right head turn (left with positive values), and the roll angle quantifies the left or right head tilt (right with positive values).

**Fig 2:**
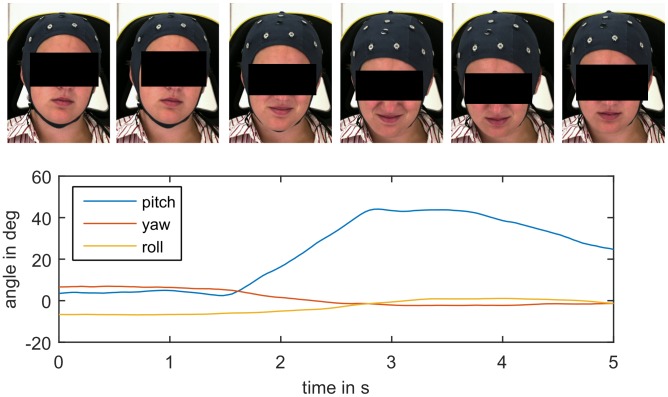
Head posture time series with corresponding video frames showing reaction to a painful heat stimulus from BioVid dataset (high temperature plateau lasting from second 0 to 3.5). The subject moves her head downwards and followed by a little upward movement, which is reflected by the increase and following decrease of the pitch angle.

For all three datasets, the subjects’ head poses were measured by the IntraFace face tracking software [45]. It measures the three orientation angles of the head (pitch, yaw, and roll, see Fig 1) relative to camera for each video frame, i.e. for each single picture of the videos, in degrees (DEG, range -180° to +180°). A whole video can be summarized by the time series of the three angles (see Fig 2 for an example). The videos in the UNBC dataset were recorded from a slight side view. To compensate for that, we corrected all yaw angles of this dataset by subtracting their mean value.

On the BioVid dataset, we tested the agreement between the used pose measurement (IntraFace software) and the alternative measurement method of Niese et al. [46]. Pearson’s correlation coefficients were 0.89, 0.94, and 0.94 for pitch, yaw, and roll angles respectively. We also calculated the single-score absolute agreement intraclass correlation coefficient (ICC) [47], which is widely used to assess inter-rater-reliability, and obtained 0.89, 0.82, and 0.87 respectively. According to the often quoted guidelines by Cicchetti [48], these numbers show an excellent level of inter-rater-reliability. Further, the mean absolute errors were 3.3°, 5.2°, and 2.1° degrees respectively, which indicate good head pose measurement accuracy [22]. Similar agreement had been found between measurements of Niese’s method and third method on another dataset by Werner et al. [39]. By agreement between IntraFace and Niese’s method, IntraFace demonstrates a high degree of concurrent validity. Further, visual review of many time series, as e.g. in Fig 2, revealed good face validity of the measurement instruments. We decided to use the IntraFace method for the subsequent analyses, because the other methods rely on 3-dimensional scans, which are not available for the UNBC dataset. In the BioVid dataset about 1% of the videos were excluded from the analyses (see Table 1), because participants left the camera’s field of view (which led to missing data) or manual review revealed obvious measurement errors.

We measured the head movement by means of the angular velocity, i.e. by differentiating the time series of angular displacements. The differentials were estimated through a Savitzky–Golay filter [49, 50] with a cubic function and a time window of 13 data points. The filter is known to improve the velocity approximation, but cannot calculate meaningful estimates for the beginning and the end of the time series. Thus, 12 data points (0.5 seconds) of each video were omitted from the analyses.

### Analysis of head posture summary statistics

We summarized the postures of each video with two statistics. The mean and the range of each orientation angle (pitch, yaw, and roll) were calculated for each of the videotaped trials. Whereas the mean measures the central tendency of the occurring poses, the range measures the difference between the most extreme angles during the whole video and captures the variability of poses independently of the duration of their occurrence. The variables were evaluated with single-factor analyses of variance (painful vs. control trials) on each dataset. We consider *p* ≤ 0.05 to be statistically significant and apply Bonferroni correction to avoid the multiple testing problem. For the BP4D dataset we compute post-hoc tests of the painful trial vs. each of the control trial types and also apply Bonferroni correction.

### Analysis of head movement summary statistics

Similar to the head postures, we summarized the head movement during each video by statistics. For each, the pitch, yaw, and roll velocity time series, we calculated the mean of the value and the mean of the magnitude. The mean value of the velocity describes the dominating movement direction. The mean of the magnitude summarizes the movement speed regarding the respective rotation axis independent of direction. We conduct analyses of variance, post-hoc analyses, and apply Bonferroni correction as in the previous section.

### Analysis of specific head posture occurrences

We looked into the occurrence of specific postures during painful and non-painful trials. For this purpose, we partitioned the occurring head postures of each dataset based on the means and standard deviations of angles. Each posture was assigned to one of three groups per angle: low value, i.e. less than mean minus standard deviation, high value, i.e. greater than mean plus standard deviation, or medium value, i.e. in between the two other groups. This way, each dataset was subdivided into 27 postures (three pitch times three yaw times three roll). Next, we counted the trial videos, in which the specific head postures occurred. For each dataset, we considered the eight postures that occurred most frequently among the pain trials. Two-sided Binomial tests were applied to compare the occurrences during pain with each of the control trial categories (with Bonferroni correction). To illustrate the posture groups, we calculated the mean posture of all frames that were assigned to the specific posture group and rendered the resulting posture using a three dimensional computer graphics head model.

### Head movement cluster analysis

The videos in the BioVid dataset had been synchronized with the pain stimulation, i.e. all videos are of equal length and the pain trial videos start one second after the applied stimulus temperature reaches the plateau. We exploited these properties for movement analysis using principal component analysis (PCA) of the time series, similar to [51]. First, we smooth the time series of the posture angles using the Savitzky–Golay filter [49, 50] (see above). Next, we subtract the mean pitch, yaw, and roll angle form the respective time series of each trial to focus on the movement rather than on postures. The resulting time series of pitch, yaw, and roll angles were concatenated to form one observation vector per trial. We applied PCA to reduce dimensionality and kept 90% of the variance, i.e. only considered the scores of the first few principal components.

We clustered the observations regarding these scores following the method applied by Kunz and Lautenbacher [52] and Rovniak et al. [53], i.e. a two step clustering procedure: In the first step, agglomerative clustering was performed using Ward’s method [54] with the Euclidian distance, which grouped the observations in a hierarchy. The number of clusters was determined through the method of Mojena [55, 56] and the cluster memberships were determined from the hierarchy accordingly. In the second steps, the observations were clustered again with the non-hierarchical k-means method using the cluster means of the first step as initial seed points. The two-step clustering allows verifying of the cluster solutions [57]; for this we calculated the agreement between the cluster memberships revealed by both methods.

Based on the second step’s cluster membership and the original time series, we calculated the mean movement across each cluster for visualization. Further, the frequencies of pain trials and no-pain trials were counted in each cluster. These frequencies were analyzed with a two-tail binomial test per cluster (with Bonferroni correction).

## Results

### Analysis of head posture summary statistics

Table 2 reports the mean and standard deviations of the posture summary statistics (“mean” and “range” for each head orientation angle) as well as the results of analyses of variance. In the BioVid dataset, the range of all three posture angles increased significantly with pain (*p* < 0.001): pitch range increased by 68% with *F*(1,3429) = 208.5, yaw range by 37% with *F*(1,3429) = 43.8, and roll range by 46% with *F*(1,3429) = 58.7. The mean yaw angle differed significantly with *p* < 0.001, *F*(1,3429) = 12.2; the mean pitch angle increased with pain by 12%, but did not reach significance level after Bonferroni correction: *p* = 0.049, *F*(1,3429) = 3.8.

**Table 2:** Head posture: Summary statistics of orientation angles (in DEG). Pitch, yaw, and roll angles of each trial video sequence were summarized by their respective mean and range. For each dataset (BioVid, UNBC, and BP4D) and statistic (columns) we report mean and standard deviation, M (SD), of pain and control trials as well as the *p*-value of the respective analysis of variance (rows). Significant differences are highlighted in bold.

	Pitch angle		Yaw angle		Roll angle	
	mean	range	mean	range	mean	range
**BioVid**						
Pain	5.5 (0.2)	8.4 (0.2)	-1.0 (0.2)	8.1 (0.3)	-0.4 (0.1)	3.5 (0.1)
No pain	4.9 (0.2)	5.0 (0.1)	0.0 (0.2)	5.9 (0.2)	-0.4 (0.1)	2.4 (0.1)
	*p* = 0.049	***p* < 0.001**	***p* < 0.001**	***p* < 0.001**	*p* = 0.877	***p* < 0.001**
**UNBC**						
Affected limb (pain)	9.8 (0.7)	16.8 (0.8)	0.0 (0.7)	15.6 (0.9)	2.3 (0.5)	11.9 (0.7)
Unaffected limb	10.6 (0.7)	13.4 (0.7)	1.1 (1.0)	17.2 (1.1)	2.2 (0.5)	10.8 (0.6)
	*p* = 0.422	*p* = 0.003	*p* = 0.350	*p* = 0.273	*p* = 0.803	*p* = 0.241
**BP4D**						
T6 (Pain)	3.9 (1.3)	21.4 (2.3)	1.0 (0.5)	18.2 (1.8)	-0.6 (0.5)	8.8 (1.1)
T1 (Amusement)	2.8 (1.2)	21.2 (1.8)	1.0 (0.4)	11.1 (1.3)	0.4 (0.5)	9.4 (1.3)
T2 (Sadness)	3.3 (0.9)	9.5 (1.8)	-3.4 (0.5)	6.6 (0.7)	-1.3 (0.6)	4.1 (0.5)
T3 (Surprise)	3.3 (1.0)	20.6 (2.2)	-2.6 (0.5)	13.8 (2.3)	-0.5 (0.4)	7.3 (0.9)
T4 (Embarrassment)	3.0 (1.0)	33.7 (3.1)	1.1 (0.4)	18.6 (2.3)	0.0 (0.5)	16.1 (2.2)
T5 (Fear)	4.1 (1.3)	25.2 (2.4)	0.3 (0.5)	14.9 (3.0)	-0.2 (0.4)	8.6 (1.6)
T7 (Anger)	2.9 (1.1)	21.5 (1.8)	2.7 (0.6)	15.4 (1.6)	0.0 (0.6)	11.4 (1.0)
T8 (Disgust)	4.9 (1.2)	18.6 (2.1)	-0.8 (0.5)	10.9 (1.5)	-0.2 (0.4)	5.3 (0.7)
	*p* = 0.890	***p* < 0.001**	***p* < 0.001**	***p* < 0.001**	*p* = 0.359	***p* < 0.001**

Pitch angle: positive / negative is lowered / raised head

Yaw angle: positive / negative is turned left / right

Roll angle: positive / negative is tilted right / left

mean: measures the central tendency of posture angles occurring during a trial

range: measures the difference between the most extreme posture angles during a trial and captures the variability of poses independently of the duration of their occurrence

In the UNBC dataset, we found no significant effects. Pitch range is 25% higher for the more painful trials, but the difference is not significant after Bonferroni correction: *p* = 0.003, *F*(1,198) = 8.8.

In the BP4D dataset, there were significant effects for all range statistics (*p* < 0.001): pitch range with *F*(7,320) = 9.3, yaw range with *F*(7,320) = 4.4, and roll range with *F*(7,320) = 8.7. Further, the mean yaw angle differed significantly with *p* < 0.001, *F*(7,320) = 17.9. Post-hoc test results (between pain and control trials) were significant in the following cases. Pitch range: T6 (Pain) vs. T2 (Sadness) with *p* < 0.001, *F*(1,80) = 16.7; T6 (Pain) vs. T4 (Embarrassment) with *p* = 0.002, *F*(1,80) = 10.3. Yaw range: T6 (Pain) vs. T1 (Amusement) with *p* = 0.002, *F*(1,80) = 10.2; T6 (Pain) vs. T2 (Sadness) with *p* < 0.001, *F*(1,80) = 37.6; T6 (Pain) vs. T8 (Disgust) with *p* = 0.002, *F*(1,80) = 10.1. Roll range: T6 (Pain) vs. T2 (Sadness) with *p* < 0.001, *F*(1,80) = 15.0; T6 (Pain) vs. T4 (Embarrassment) with *p* = 0.003, *F*(1,80) = 9.2. Yaw mean: T6 (Pain) vs. T2 (Sadness) with *p* < 0.001, *F*(1,80) = 36.1; T6 (Pain) vs. T3 (Surprise) with *p* < 0.001, *F*(1,80) = 25.1.

### Analysis of head movement summary statistics

Table 3 reports the mean and standard deviations of the movement summary statistics (“mean of velocity value” and “mean of velocity magnitude” for each head orientation angle) as well as the results of analyses of variance. In the BioVid dataset, we found significant differences for the mean of pitch velocity values with *p* < 0.001, *F*(1,3429) = 96.2, and the mean of yaw velocity values with *p* < 0.001, *F*(1,3429) = 55.6. Further, the velocity magnitudes of all three angles were significantly higher during pain (*p* < 0.001): pitch by 46% with *F*(1,3429) = 238.5, yaw by 20% with *F*(1,3429) = 31.3, and roll by 31% with *F*(1,3429) = 51.2.

**Table 3:** Head movement: Summary statistics of angular velocities (in DEG/s). Pitch, yaw, and roll velocity of each trial video sequence were summarized by their respective mean value and mean of magnitude. For each dataset (BioVid, UNBC, and BP4D) and statistic (columns) we report mean and standard deviation, M (SD), of pain and control trials as well as the *p*-value of the respective analysis of variance (rows). Significant differences are highlighted in bold.

	Pitch velocity: mean of …		Yaw velocity: mean of …		Roll velocity: mean of …	
	value	magnitude	value	magnitude	value	magnitude
**BioVid**						
Pain	3.2 (0.3)	37.4 (0.7)	-4.0 (0.5)	31.3 (0.7)	0.1 (0.2)	13.6 (0.3)
No pain	-0.5 (0.2)	25.6 (0.4)	0.2 (0.3)	26.1 (0.6)	0.0 (0.1)	10.4 (0.3)
	***p* < 0.001**	***p* < 0.001**	***p* < 0.001**	***p* < 0.001**	*p* = 0.614	***p* < 0.001**
**UNBC**						
Affected limb (pain)	1.4 (0.8)	53.7 (2.3)	0.2 (0.9)	42.3 (2.0)	1.7 (0.8)	26.1 (1.2)
Unaffected limb	-1.7 (1.3)	52.9 (2.4)	-3.2 (2.3)	57.6 (3.0)	3.9 (1.3)	36.6 (2.1)
	*p* = 0.034	*p* = 0.814	*p* = 0.145	***p* < 0.001**	*p* = 0.129	***p* < 0.001**
**BP4D**						
T6 (Pain)	2.1 (0.8)	47.8 (3.3)	2.1 (0.8)	27.1 (1.4)	0.1 (0.4)	13.4 (1.2)
T1 (Amusement)	0.9 (0.5)	59.1 (3.6)	0.2 (0.2)	27.2 (1.7)	0.4 (0.2)	16.5 (1.4)
T2 (Sadness)	0.0 (0.1)	25.3 (1.8)	0.0 (0.0)	20.5 (0.7)	-0.1 (0.1)	7.2 (0.3)
T3 (Surprise)	5.9 (1.6)	54.5 (4.0)	1.1 (1.0)	37.0 (3.0)	0.1 (0.5)	16.5 (1.1)
T4 (Embarrassment)	1.2 (0.5)	71.2 (5.0)	-0.3 (0.1)	40.6 (3.5)	0.3 (0.2)	26.8 (2.9)
T5 (Fear)	0.8 (0.3)	52.0 (3.3)	0.2 (0.2)	27.4 (2.3)	-0.1 (0.1)	13.1 (1.1)
T7 (Anger)	0.2 (0.3)	49.4 (2.9)	0.3 (0.2)	34.2 (2.9)	-0.0 (0.1)	18.8 (1.8)
T8 (Disgust)	7.1 (1.8)	62.7 (4.5)	-2.0 (0.8)	36.8 (4.5)	-0.9 (0.4)	16.4 (1.6)
	***p* < 0.001**	***p* < 0.001**	***p* < 0.001**	***p* < 0.001**	*p* = 0.081	***p* < 0.001**

Pitch velocity value: positive / negative is lowering / raising head

Yaw velocity value: positive / negative is turning left / right

Roll velocity value: positive / negative is tilting right / left

mean of the velocity value: describes the dominating movement direction

mean of the velocity magnitude: summarizes the movement speed regarding the respective rotation axis independent of direction

In the UNBC dataset, analyses of variance yielded significant effects for yaw and roll velocity magnitude (*p* < 0.001), yaw with *F*(1,198) = 18.8, and roll with *F*(1,198) = 20.4. In contrast to the BioVid dataset, movement speed is lower for the more painful trials. Similarly to BioVid, the pitch velocity value was positive, i.e. the head movement tendency during pain was downwards, but the effect was not significant after Bonferroni correction.

In the BP4D dataset, significant effects were observed for pitch and yaw velocity value (*p* < 0.001): pitch with *F*(7,320) = 8.5 and yaw with *F*(7,320) = 4.4. Further, we found effects for all angles’ velocity magnitudes (*p* < 0.001): pitch with *F*(7,320) = 13.5, yaw with *F*(7,320) = 6.1, and roll with *F*(7,320) = 12.2. Post-hoc analyses found the following significant differences. Pitch velocity magnitude: T6 (Pain) vs. T2 (Sadness) with *p* < 0.001, *F*(1,80) = 35.3; T6 (Pain) vs. T4 (Embarrassment) with *p* < 0.001, *F*(1,80) = 15.1. Yaw velocity value: T6 (Pain) vs. T8 (Disgust) with *p* < 0.001, *F*(1,80) = 12.8. Yaw velocity magnitude: T6 (Pain) vs. T2 (Sadness) with *p* < 0.001, *F*(1,80) = 16.7; T6 (Pain) vs. T3 (Surprise) with *p* = 0.004, *F*(1,80) = 8.9; T6 (Pain) vs. T2 (Embarrassment) with *p* < 0.001, *F*(1,80) = 12.9. Roll velocity magnitude: T6 (Pain) vs. T2 (Sadness) with *p* < 0.001, *F*(1,80) = 26.5; T6 (Pain) vs. T2 (Embarrassment) with *p* < 0.001, *F*(1,80) = 18.3.

### Analysis of specific head posture occurrences

**Fig 3:**
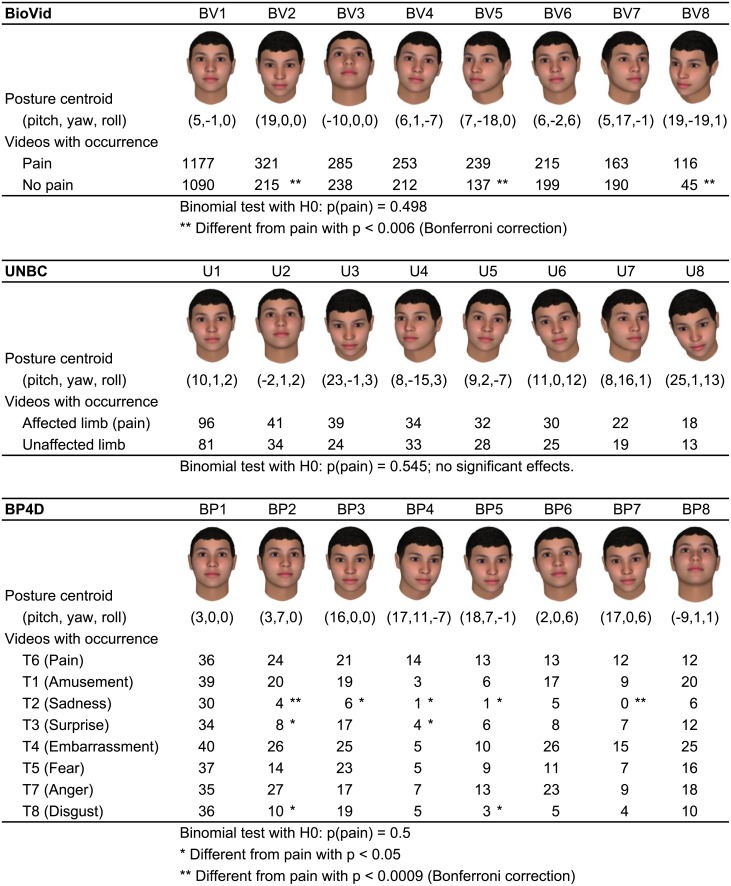
Specific head posture’s occurrence counts and significance test results. Each dataset was subdivided in 27 postures. The 8 postures that occurred most frequently among pain trials were considered for comparing the frequency of occurrences in pain and control trials with binomial tests. The figure illustrates the 8 postures per dataset and lists the occurrence frequencies in the trial categories. Significant differences are marked by asterisks.

As described in the methods section we subdivided each dataset in 27 postures and analyzed the 8 postures that occurred most frequently among pain trials. Fig 3 shows the postures, their occurrence frequencies, and results of the binomial tests. For all three datasets, the most frontal posture (BV1, U1, BP1) occurred most often during pain, but also during the control trials, i.e. it is not specific to pain. Among the next most frequent postures, there were several variations of the pitch angle: the lowered head (BV2, U3, BP3) and the raised head (BV3, U2). The lowered head occurred significantly more often during pain than during the non-painful trials in the BioVid dataset (BV2). It is also more frequent for UNBC (U3) and BP4D (BP3), but the difference does not reach significance level there. Other postures that occur significantly more often during pain in BioVid are BV5 and BV8, i.e. turning to the right as well as turning right and downwards. In the BP4D dataset, we found two postures that occurred significantly more often during pain than during T2 (sadness): the head turned left (BP2) and lowered and tilted right (BP7). Several other marked differences (labeled with single *) were not significant after Bonferroni correction.

### Head movement cluster analysis

**Fig 4:**
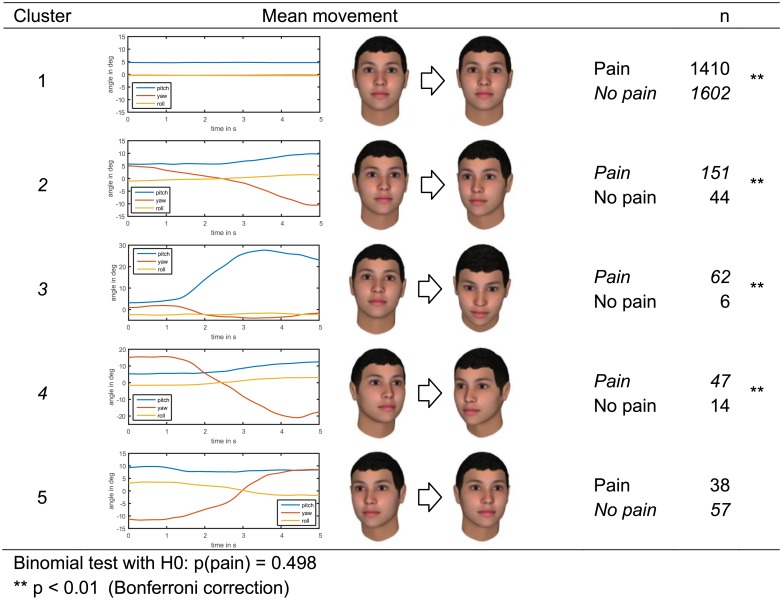
Head movement clusters in the BioVid dataset with number of pain and control trials falling into the cluster. Clusters are illustrated by their mean movement. Significant differences (according to the conduced binomial tests) are marked by asterisks.

To analyze head movement further, we applied cluster analysis on the BioVid dataset as described in the methods section. The cluster analysis yielded five clusters and a high agreement of 95.3% between the hierarchical and k-means method. Fig 4 illustrates the clusters and the corresponding frequencies of pain and control trials. Cluster 1, which was the largest cluster, contained no movement (and also some a-typical movements like raising the head which are not visible in the mean). This movement type occurred significantly less often during pain (*p* < 0.001). Cluster 2, a moderate head turn to the right with a slight downward movement, occurred significantly more often during pain (*p* < 0.001). The same applied for cluster 3, a strong downward movement, and cluster 4, a strong turn to the right with a slight downward movement. In cluster 5, which was characterized a head turn to the left, pain trials were in minority, but the difference was not significant.

## Discussion

We found significant differences in head movements and postures (HMP) between pain and control trials in all three datasets. Results were similar for the BioVid and BP4D dataset. In both, HMP during pain tended to be oriented downwards and towards the stimulus side, i.e. towards the right arm for the BioVid dataset and towards the left arm for the BP4D dataset.

The strong evidence for downward orientation during pain experience in these datasets, particularly in the BioVid dataset, is in line with the research of Walsh [58], in which “head averted”, “gaze downward”, and “forward body lean” were among the key components of the body postures for pain as performed by actors. Explanation for this head orientation can be diverse. First, forward bended head postures relief neck muscle from pain induced activity, which is part of the pain response in many pain conditions [59, 60]. Additionally, the forward bending may be part of a depressive response [31, 32], indicating the withdrawal from the pain inducing situation and social communication. From an evolutionary point of view [61] downward head bending can also be seen as a genetically determined behavior pattern, which has been beneficial for survival as a lowered head can help to protect throat and face and reduce overall attack surface further. With such a submissive posture [27], a person in pain might look less threatening to a potential attacker [58], so that he will refrain from attacking the opponent. Finally, the bowed head is part of the fetal position, which is a protective and comforting posture and is listed as one item in the PACSLAC pain assessment tool [17].

HMP towards the stimulus side may be related to the focus of attention. Looking at the site of pain can precede or even initiate touching or rubbing the affected area, which is a typical item in behavioral scales [13, 15, 62]. Alternatively, it is probable that subjects are turning their attention to the pain stimulation due to neural pathways that project into the limbic areas of the brain leading to negative emotion, which may induce behavioral responses. In addition, the electrophysiological data suggest that this effect is mediated by a stimulus-driven process, in which somatic threat detectors located in the dorsal posterior insula activate the medial and lateral prefrontal cortex areas involved in reorienting attention towards the painful target [63].

We also found high movement range and faster movement in painful situations. This is consistent with several items in behavioral pain scales, e.g. “vigorous movements including torso and head” in the COMFORT scale [11], as well as activity and restlessness described in FLACC [12] and CNPI [13], for instance.

### Comparison of datasets

The examined datasets differed in several aspects. Among the three datasets, BioVid provided the strongest evidence for HMP being part of pain behavior. It was the largest dataset in terms of subjects and trials, i.e. some tests in the other datasets may have failed to reach significance due to sample size. The participants recorded in the BioVid dataset were explicitly allowed to move their head freely in a sitting position. For the other datasets, it is unknown whether they got instruction to look towards the camera, which is not uncommon for datasets that mainly target facial expression analysis. Further, the BioVid study was designed to minimize social influences and avoid interaction, i.e. the study participant was alone during data collection (but had the option to call for help or stop the experiment). In contrast, UNBC and BP4D both included social interaction, which might be a confounding variable for analyzing HMP. For instance, in UNBC the patients sometimes talked to the experimenter, which was probably accompanied by moving the head to look at her. In the BP4D dataset, most of the tasks that were used to elicit emotions involved social interaction with the experimenter. This might have induced interaction-related HMP, which could not be isolated from emotion-related HMP. In BP4D, significant differences were mainly found between pain and sadness. The sadness task involved watching a video documentary. So the participant focused the attention on the screen resulting in less head movement than during pain and the other tasks. Sadness generally tends to be associated with less movement than other emotions, e.g. Walbott found that “In sadness, movements were less expansive (i.e., quite small in terms of space), whereas in anger, and especially in surprise, movements were more expansive.” [64]

In contrast to BP4D and UNBC, the control trials in the BioVid dataset were resting periods, i.e. there was no social interaction, no emotion elicitation, and no other events guiding the visual attention. In this case, pain and control trials could be distinguished with quite simple measures of head activity. When social interactions or emotions were involved (in BP4D and UNBC), HMP were more complex and could not be distinguished with the used measures in most cases. With current methods, detailed analysis is difficult when time series are not synchronized with stimulation events and differ in length. E.g. the head movement cluster analysis could not be applied to the BP4D and UNBC dataset. More research in human movement analysis may help to find better methods and measures to analyze posture time series. For instance, long sequences that consist of multiple behavioral actions and reactions could be split into meaningful subsequences to analyze them individually.

The HMP that we observed in the UNBC dataset differ from the patterns that we found in BioVid and BP4D. We see two possible reasons: First, the UNBC study participants self-identified as having pain problems (in contrast to the healthy participants of the other studies). Many of them were not pain-free in control trials. Further, some participants probably suffered from chronic pain, which can be associated with altered behavioral response to pain (compared to acute pain). A second possible reason is the different pain site, which is the shoulder (instead of the forearm). In the more painful trials, we observed reduced magnitudes of yaw and roll velocity, i.e. slower and less side movements compared to the less painful trials. This might be related to guarding or shoulder muscle tension during pain, similar to the results of Traue et al., who found that headache with muscular symptoms can be associated with reduced head motion [36]. We also found that the pitch angle range increased with pain (more down- and upward movement, as in BioVid), but the effect was not significant after Bonferroni correction, which might be a sample size issue. Further, there are two properties of the dataset that may have interfered with the HMP analysis. First, the pain stimulation (range-of-motion test) involved shoulder movements that possibly influenced the head movements. Second, the control trials were less painful, but not pain-free. So they may also contain some pain behavior.

### Strengths and limitations

Our study is one of the first analyses of HMP occurring during pain. We applied latest computer vision technology to measure the head posture and movements quantitatively. Compared to coding by humans (as done in most previous works), this facilitates higher accuracy and analysis of larger datasets. We studied three datasets with different pain modalities, populations, and control trials. This is a major strength, as we were able to identify some common HMP. But it also leads to limitations of our study, since we used existing datasets and could not control for all confounding variables, such as social interaction or movement caused by the pain stimulation itself (range-of-motion test in UNBC). More research is needed to identify the factors influencing HMP. Further, we used datasets with active pain stimulation, which differs from clinical pain conditions and chronic pain. Thus, results are not directly transferable to relevant clinical populations, such as people with dementia. Generally, HMP should be studied in more clinical populations to evaluate their relevance for clinical pain assessment.

## Conclusions

Our analysis found significant differences in head movements and postures (HMP) between pain trials and control trials. Most notably, pain was accompanied by HMP that tend to be oriented downwards or towards the pain site. Further, we found differences in movement range and speed.

The related domain of facial pain expression has been a research focus and fruitfully under debate for many years [1, 61, 65, 42]. We observed that HMP often co-occur with facial expression (see Fig 2), but they also occur alone. Results from Werner et al. [39, 38, 5] suggest that HMP can complement facial expression (and other behavioral pain markers) as an additional cue. In these works, pain intensity could be predicted correctly in more of the unseen cases if HMP information was combined with facial expression than from facial expression alone. Further, these and other publications show promising results for future automatic pain monitoring systems; those have potential to reduce workload associated with pain assessment, provide continuous assessment, and might facilitate more objective assessment. Next to the automatic analysis of facial expression, which has already been successfully tested in a clinical context [8], these systems could incorporate HMP and also body gestures. Similar automated behavior analysis methods have shown potential in depression screening, diagnosis and research [10].

HMP might be also useful for pain assessment done by the clinical practitioner if we are able to describe HMP more precisely and develop a better understanding of their communicative role or their correlate of pain intensity as well as quality. Social interaction influences head movements, but in contrast to the used computer-based methods a human observer should be able to identify interaction related movements and exclude them from his assessment.

Overall, the results suggest that HMP should be considered for pain assessment and research, as they may be gestures with symbolic pain-related meaning. Possibly, HMP might improve pain management whenever behavior is assessed, especially in nonverbal individuals such as infants or patients with dementia. However, in advance more research is needed to investigate HMP in those populations.
